# An alkaline protease from *Bacillus cereus* NJSZ-13 can act as a pathogenicity factor in infection of pinewood nematode

**DOI:** 10.1186/s12866-022-02752-2

**Published:** 2023-01-10

**Authors:** Liangliang Li, Yufeng Sun, Fengmao Chen, Dejun Hao, Jiajin Tan

**Affiliations:** 1 College of Forestry, Nanjing Forestry University, Nanjing, People’s Republic of China; 2 Institute of Biology Co., Ltd., Henan Academy of Sciences, Zhengzhou, People’s Republic of China

**Keywords:** Endophytic bacteria, *Bursaphelenchus xylophilus*, Extracellular protease, Nematicidal activity, *Bacillus cereus*

## Abstract

Endophytic bacteria are an important biological control for nematodes. We isolated the nematicidal *Bacillus cereus* NJSZ-13 from healthy *Pinus elliottii* trunks. Bioassay experiments showed killing of all tested nematodes by proteins from the NJSZ-13 culture filtrate within 72 h. Degradation of the nematode cuticles was observed, suggesting the action of extracellular bacterial enzymes. The responsible protease was purified by ammonium sulfate precipitation, hydrophobic interaction chromatography, ion-exchange chromatography, and SDS-PAGE. The protease had a molecular weight of 28 kDa and optimal activity at 55 °C and pH 9, indicating an alkaline protease. The study suggests the potential for using this *B. cereus* NJSZ-13 strain protease to prevent pinewood nematode infection.

## Introduction

*Bursaphelenchus xylophilus*, the pinewood nematode, causes pine wilt. Due to the severity of its effects, the pinewood nematode is considered as an important quarantine pest in many countries. Pine wilt disease is dependent on a variety of factors, including the host tree, long-horned beetles, fungi, bacteria, environmental factors, as well as *B*. *xylophilus* infection [1]. The mechanism of pathogenesis remains unknown, and thus prevention and management are of global concern. In response to the appeal of green agriculture in the current environmentally friendly social atmosphere, traditional chemical controls are no longer considered adequate for the management of the pinewood nematode. Chemical pesticides are undesirable due to their adverse effects on the environment and ecological balance, and also because extensive use may result in the development of resistance by the target species leading to diminishing control efficiency [2, 3]. Biological means are, therefore, increasingly seen as alternatives for effective pest control.

Biological controls aim to reduce the nematode population by introducing predators, parasites, or toxins produced by such natural enemies. Several efficient biological control agents against nematodes, many derived from nematophagous fungi, are currently available [4, 5]. Bacterial biocontrol of plant-parasitic nematodes has recently become an important topic in biological control with the identification of numerous suitable bacteria and the documentation of their actions. Specifically, “*Bacillus thuringiensis* [6]”, *Pasteuria penetrans* [7], *Pseudomonas* spp. [8], and plant growth-promoting rhizobacteria [9] have been shown to be effective for nematode control. The value of such bacterial biological controls is gradually being recognized. Previously, most biological control agents were isolated from the rhizospheric soil. However, these soil microorganisms are not only sensitive to external conditions but also less competitive among soil, rhizospheric, or phyllospheric organisms. This has greatly affected their practical value [10]. In contrast, endophytic bacteria of plants have many advantages as biological control agents. Endophytic bacteria are distributed in different tissues of plants; they have abundant nutrients; they are protected by plant tissues and are sheltered from intense sunlight, UV radiation, wind, and rain, meaning that their environment is very stable [11]. Therefore, endophytic bacteria are often more effective as biological controls than other bacteria.

Plant endophytic bacteria and pinewood nematodes both live in plant tissues. It can thus be assumed that after endophytic bacteria with nematicidal activity are inoculated into the susceptible parts of pine trees, the bacteria can directly reach the tissues infected by the pinewood nematodes. This can not only control the nematodes but also assist the growth-promoting activities of the endophytic bacteria [11]. We have previously described an endophytic bacterium from the interior of a healthy *Pinus elliottii* tree in the Nanjing Zhongshan Botanical Garden; this was identified as *B. cereus* strain NJSZ-13 [12]. In this study, histological observation showed that the extracellular proteases from strain NJSZ-13 degrading nematode cuticles were involved in the process of penetrating the epidermis and finally digest them. Subsequently, the cuticle-degrading enzyme was chromatographically purified and characterized.

## Materials and methods

### Culturing of strain NJSZ-13 and pinewood nematode

The *B. cereus* strain NJSZ-13 was grown in NB medium (1% peptone, 0.3% beef extract, 0.5% NaCl, pH 7.2 ± 0.2) at 28 °C on a rotary shaker at 200 rpm. The test species was the pinewood nematode *B. xylophilus* which was cultured in *Botrytis cinerea* for 5–7 days at 25 °C and harvested using the Baermann funnel technique [13]. An nematode suspension (5 nematodes/μL) in sterile PBS (pH 7.4) was used as working stock.

### Bioassay

The bioassay was performed as previously described [14]. Briefly, the NA medium (1% peptone, 0.3% beef extract, 0.5% NaCl, 2% agar, pH 7.2 ± 0.2) plates were covered with axenic cellophane paper to prevent movement of the nematodes into the medium. The bacteria were placed on the cellophane and cultivated in an incubator at 28 °C. When the entire plate was covered by the bacterial colony, 100 μL of the nematode suspension (containing about 500 nematodes) was transferred to the plate center. The plates were each divided into 20 sections, and every 24 h, five sections were randomly selected and the nematode mortality assessed using a light dissecting microscope. Experiments were conducted in triplicate, and at least 30 nematodes were observed each time. Non-pathogenic bacteria (*Escherichia coli*, ATCC 25922) were used as a control.

Nematodes treated with strain NJSZ-13 were picked up and gently mounted in sterile PBS containing 1.42 g/L Na_2_HPO_4_, 0.27 g/L KH_2_PO_4_, 0.2 g/L KCl, and 8 g/L NaCl, pH 7.4 before examination under light microscopy and scanning electron microscopy (SEM). If the nematodes did not move when examined under the light microscope or if they did not respond to light tapping with a needle, they were considered dead. The percentage of dead nematodes was described as the mortality rate.

### Extraction of extracellular proteins from NJSZ-13

The strain NJSZ-13 was inoculated into a 250-mL Erlenmeyer flask containing 100 mL NB medium and incubated at 28 °C on a rotary shaker (200 rpm) for 4 days. The culture solution was centrifuged at 8500 *g* and 4 °C for 15 min, the supernatant retained, and sufficient (NH_4_)_2_SO_4_ added to 100% saturation. After standing at 4 °C overnight, the solution was centrifuged at 5500 *g* at 4 °C for 30 min and the precipitate was dissolved in 10 mL of sterile PBS (pH 7.4). The solution was loaded into a dialysis bag with a cut-off molecular weight of ~ 8000–15,000 Da. The dialysis bag was then immersed in 20 volumes of phosphate buffer, changed four times with each dialysis lasting approximately 3 h. The resulting component was the crude extracellular protein extract of the strain NJSZ-13. The crude extract was filtered through a 0.22-μm filter and used immediately.

### Measurement of the effects of the protein extract against the pinewood nematode

Two hundred microliters of the protein extract were placed in sterile 1.5 mL Eppendorf tubes with 200 μL of the nematode suspension (approximately 1000 nematodes) and incubated at 25 °C. Each 24 h, a 20 μL aliquot of the protein-nematode suspension (approximately 50 nematodes) was placed on a glass slide and the nematode mortality assessed under light microscopy. Experiments were conducted in parallel with five replications and were repeated three times. Controls contained sterile PBS (pH 7.4), NB medium, NJSZ-13 culture supernatant, and heat-denatured (100 °C for 15 min) protein extract.

Changes in the cuticles of the nematodes were assessed under light microscopy and SEM.

### Preparation of nematode samples for the light microscope and SEM

The treated nematodes were immersed in sterile PBS (pH 7.4). The nematodes were then observed and photographed under the light microscope (Axio Imager M2; Zeiss, Oberkochen, Germany).

For the SEM observation, the nematodes were first pre-fixed in glutaraldehyde (4%) in sterile PBS at 4 °C for 2 h then dehydrated in serial ethanol concentrations (50, 70%, two changes of 90%, and three changes of 100% ethanol for 10 min each) at room temperature. The dehydrated nematodes were then dried by a critical point drying instrument (K850; Emitech, East Sussex, UK) and sputter-coated with Au-Pd using ion-sputtering equipment (E-1010; Hitachi, Tokyo, Japan) before being examined and photographed with a “desktop SEM (NeoScope JCM-5000; Nikon, Tokyo, Japan)”.

### Protease purification

Five hundred milliliters of NJSZ-13 culture was centrifuged at 8500 *g* and 4 °C for 15 min. Sufficient (NH_4_)_2_SO_4_ was added to the supernatant to achieve 20% saturation. After standing overnight at 4 °C, the solution was centrifuged (5500 *g*, 4 °C, 30 min), and the supernatants were pooled. Then, (NH_4_)_2_SO_4_ was added to reach 40, 60, 80%, and finally 100% saturation, solutions were centrifuged as above, and the nematicidal pellet was dissolved in PBS for chromatography.

Hydrophobic interaction chromatography (HIC). The dissolved precipitate with nematicidal activity was supplemented with 1 M (NH_4_)_2_SO_4_ and applied to a HiTrap™ phenyl FF column (high sub，1 mL; General Electric Company, Boston, USA) that had been equilibrated with sterile PBS containing 1 M (NH_4_)_2_SO_4_ (pH 7.4). The bound proteins were eluted with a linear gradient of 1–0 M (NH_4_)_2_SO_4_ in PBS with a flow rate of 1 mL/min. Fractions (1 mL) were collected, dialyzed, and the protease and nematicidal activity was measured.

Ion-exchange chromatography (IEC). Fractions positive for protease and nematicidal activities were pooled and applied to a HiTrap™ Capto Q column (1 mL; General Electric Company, Boston, USA) equilibrated with sterile PBS (pH 6.0). Bound proteins were eluted with a linear gradient of 0–1 M NaCl (pH 6.0) with a flow rate of 1 mL/min. Fractions (1 mL) were collected, dialyzed, and their protease and nematicidal activities evaluated.

SDS-PAGE. Fractions positive for protease and nematicidal activity were electrophoresed by SDS-PAGE (12% running gel and 4% concentration gel). Gels were “stained with Coomassie brilliant blue R-250” for 2 h.

### Protease assays

Protease activity was measured with 1% casein solution as substrate. One hundred and twenty-five microliters of 1% casein in sterile PBS (pH 7.4) and the protease-containing sample were incubated in 1.5 mL-Eppendorf tubes at 37 °C for 10 min. The reaction was halted by the addition of 250 μL of 0.4 M trichloroacetic acid (TCA), and the solution was centrifuged at 4 °C and 8500 *g* for 15 min. The supernatant was transferred to tubes containing 2.5 mL of 0.4 M sodium carbonate and 0.5 mL Folin phenol reagent, incubated at 37 °C for 20 min, and the OD at 680 nm read in a spectrophotometer. A tyrosine-containing standard was used for calibration and the protease activity in the samples was assessed as the tyrosine increase over 1 min at 37 °C.

The protein content was measured using an enhanced BCA protein assay kit (Beyotime, Shanghai, China) with bovine serum albumin (BSA) as the standard.

### Characterization of the protease

To determine the optimum temperature for proteolytic reaction, the activity of the protease was measured by incubating the reaction mixture at 20, 30, 37, 45, 50, 55, 60, 70, 80 and 90 °C as described above. The optimum pH was measured with the “Britton Robinson universal buffer system” at pH 4.0, 5.0, 6.0, 7.0, 8.0, 9.0, 10.0, and 11.0, respectively.

Samples were incubated in the presence of different metals (Li^+^, K^+^, Na^+^, Mg^2+^, Ca^2+^, Mn^2+^, Zn^2+^, and Ba^2+^) to measure the effects of metals on the enzyme with protease activity without any metal ions set to100%.

## Results

### Nematicidal activity of strain NJSZ-13

Strain NJSZ-13 exhibited significant nematicidal behavior in the bacterial infection experiments. Within 48 h, 41.6% of the nematodes were dead, and 87.3% were dead after 72 h in the NJSZ-13-containing plates. In the negative control containing a non-pathogenic bacterium (*E. coli*), the majority was normally mobile, and the mortality of the nematodes remained 8.2% within 72 h.

**Fig. 1:**
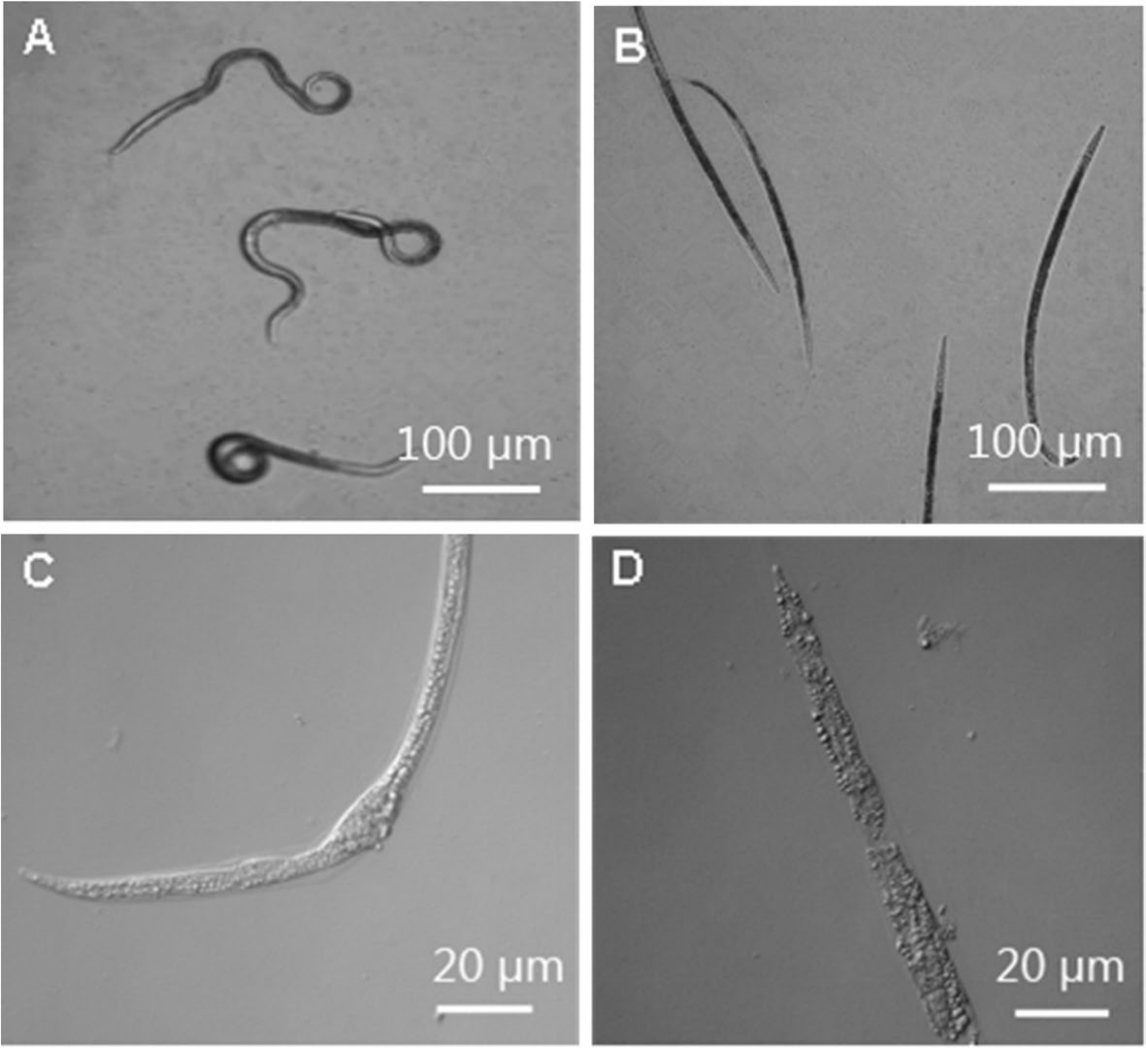
Infection of *B. xylophilus* with *B. cereus* strain NJSZ-13 seen under light microscopy: **A** Most of the control nematodes were mobile within 72 h; **B** More than 80% of nematodes were dead after 72 h; **C** Body of nematode treated with NJSZ-13 for 48 h; **D** Degradation and digestion of nematode cuticle after treatment with NJSZ-13 for 72 h

**Fig. 2:**
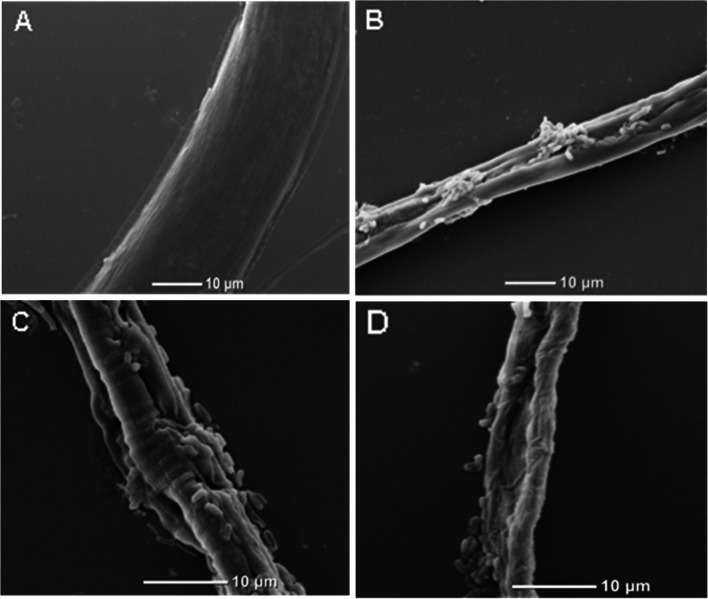
Effects of treatment with strain NJSZ-13 on *B. xylophilus* observed under SEM: **A** Smooth and intact cuticles seen in the control groups after 72 h; **B** Body of a nematode showing binding by strain NJSZ-13; **C** Cracks containing bacteria visible on the nematode surface; **D** Complete destruction of the body of a nematode

The light microscope (Fig. 1) and SEM (Fig. 2) were used to record histopathological changes and the infectious process of nematodes treated with strain NJSZ-13. Under the light microscope, degradation and destruction of the nematode cuticles was clearly visible (Fig. 1B, C, D). To observe the infectious process of the nematodes treated with NJSZ-13, the nematodes were picked up and gently mounted in sterile PBS (pH 7.4) and then were observed with the SEM. Strain NJSZ-13 first bound to the epidermis before penetrating and degrading the cuticle (Fig. 2B), leading to holes full of bacteria on the nematode surface (Fig. 2C). Later, broken nematode bodies were observed (Fig. 2D). In contrast, the nematodes in the control groups appeared normal with smooth cuticles after 72 h (Fig. 1A, and Fig. 2A).

### Toxic effect of the NJSZ-13 protein extract against the pinewood nematode

As shown in Table 1, both the culture filtrate of NJSZ-13 and its crude extracellular protein extract had nematicidal activities. The nematicidal activities of these two treatments increased with increasing treatment time during the test period. For the culture filtrate, the mortality rates of the nematodes were 16.3, 32.1 and 58.6% after 24 h, 48 h, and 72 h treatments, respectively. When nematodes were incubated in the protein extract, the mortality rates were 38.3, 72.4 and 100% after 24 h, 48 h, and 72 h, respectively. Although some nematodes treated with the PBS and NB medium died, the mortality was very low (below 6% after 72 h). The above results indicated that the strain NJSZ-13 protein extract had significant toxicity against pinewood nematodes. However, in the control with the boiled extract, the mortality of the nematodes was only 28.7% after 72 h, and the cuticles of the nematodes were not degraded. This suggested that the nematicidal material from the crude extracellular protein extract was not heat stable.

**Table 1:** The nematicidal activity of the protein extract from strain NJSZ-13 against *B. xylophilus*

Samples	Mortality of *B. xylophilus*/%(SD)		
	24 h	48 h	72 h
Culture filtrate	16.3 ± 1.2	32.1 ± 2.1	58.6 ± 0.7
Crude extracellular protein extract	38.3 ± 2.5	72.4 ± 2.8	100 ± 0
Crude extracellular protein extract, boiled	13.3 ± 0.9	19.8 ± 1.9	28.7 ± 2.6
NB medium	2.2 ± 0.1	3.5 ± 0.3	5.6 ± 1.2
PBS	1.4 ± 0.6	2.1 ± 0.2	2.8 ± 0.3

*Note*: SD represents standard deviation (n = 3)

**Fig. 3:**
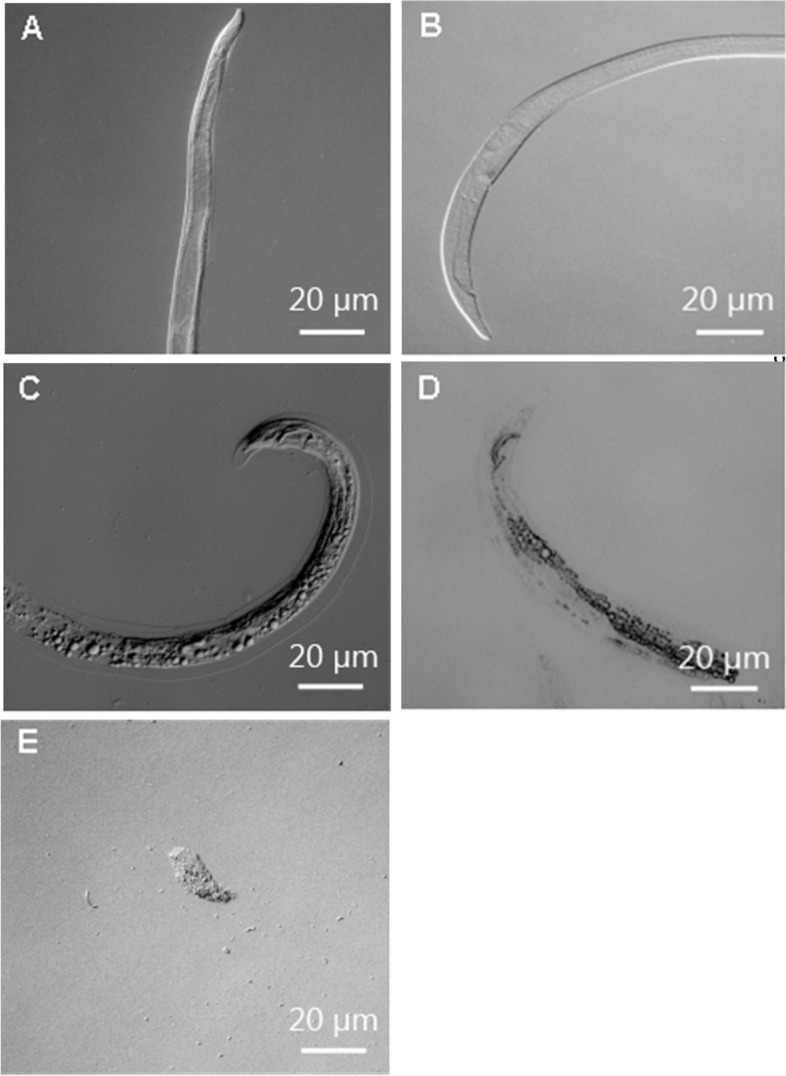
Toxicity of the NJSZ-13 protein extract against *B. xylophilus* seen under light microscopy: **A** In PBS, the nematode was mobile and alive after 72 h; **B** In NB medium, the cuticle was complete and smooth after 72 h; **C** Treatment with the protein extract damaged the cuticle within 24 h; **D** Degradation of the nematode cuticle after 48 h of treatment with the protein extract; **E** After 72 h, only cellular fragments of nematodes were visible

**Fig. 4:**
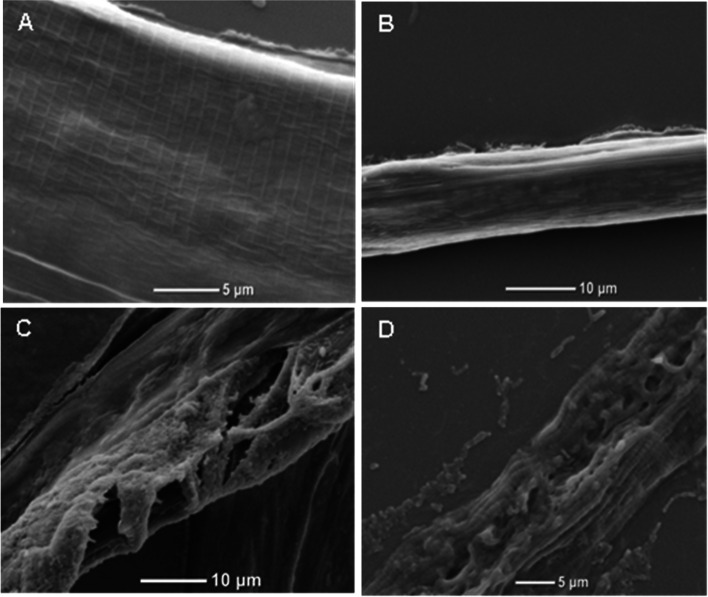
Toxicity of the NJSZ-13 protein extract against *B. xylophilus* observed under SEM: **A**, **B** Smooth surfaces in nematodes in PBS and LB medium after 72 h showing clear striae and lateral lines; **C** Scars and flaws in the cuticle visible after treatment for 48 h; **D** Exfoliation of the cuticle visible after 72 h

Cuticle destruction of the nematodes by the crude extracellular protein extract was observed with light microscopy (Fig. 3) and SEM (Fig. 4). Under the light microscope, it was observed that the mobility of the treated nematodes gradually decreased; some nematodes began moving abnormally, and other nematodes hardly showed any signs of mobility within 24 h. By 48 h, most nematodes were immobile; only a small percentage of the nematodes showed slight mobility when touched with dissecting needles. After 72 h, no nematodes showed signs of life. No signs of life were observed after the nematodes were subsequently washed with fresh water. The cuticles of over 90% of the dead nematodes were almost totally destroyed after 72 h. Initially, the cuticles of the nematodes were observed to be incomplete (Fig. 3C); afterward, the cuticles began to be degraded and digested (Fig. 3D). Finally, only cellular fragments of nematodes remained (Fig. 3E). In contrast, the nematodes treated with NB medium and PBS in the control groups appeared unaffected; no cuticle degradation was observed, and the cuticles were smooth and intact after 72 h (Fig. 3A, B).

To closely examine the cuticular changes of nematodes treated with the crude extract, the nematodes were observed under the SEM. The nematode cuticles in the control groups were smooth and complete, and distinct stripes and lateral lines were clearly visible (Fig. 4A, B). However, the cuticles of nematodes treated with the crude extract were destroyed. When treated for 48 h, large cracks and indentions appeared on the bodies of the nematodes and inclusions inside the bodies of the nematodes were discharged (Fig. 4C). After 72 h, exfoliation was visible with only the broken exocuticle remaining (Fig. 4D).

### Purification of the extracellular protease from NJSZ-13 culture filtrates

**Fig. 5:**
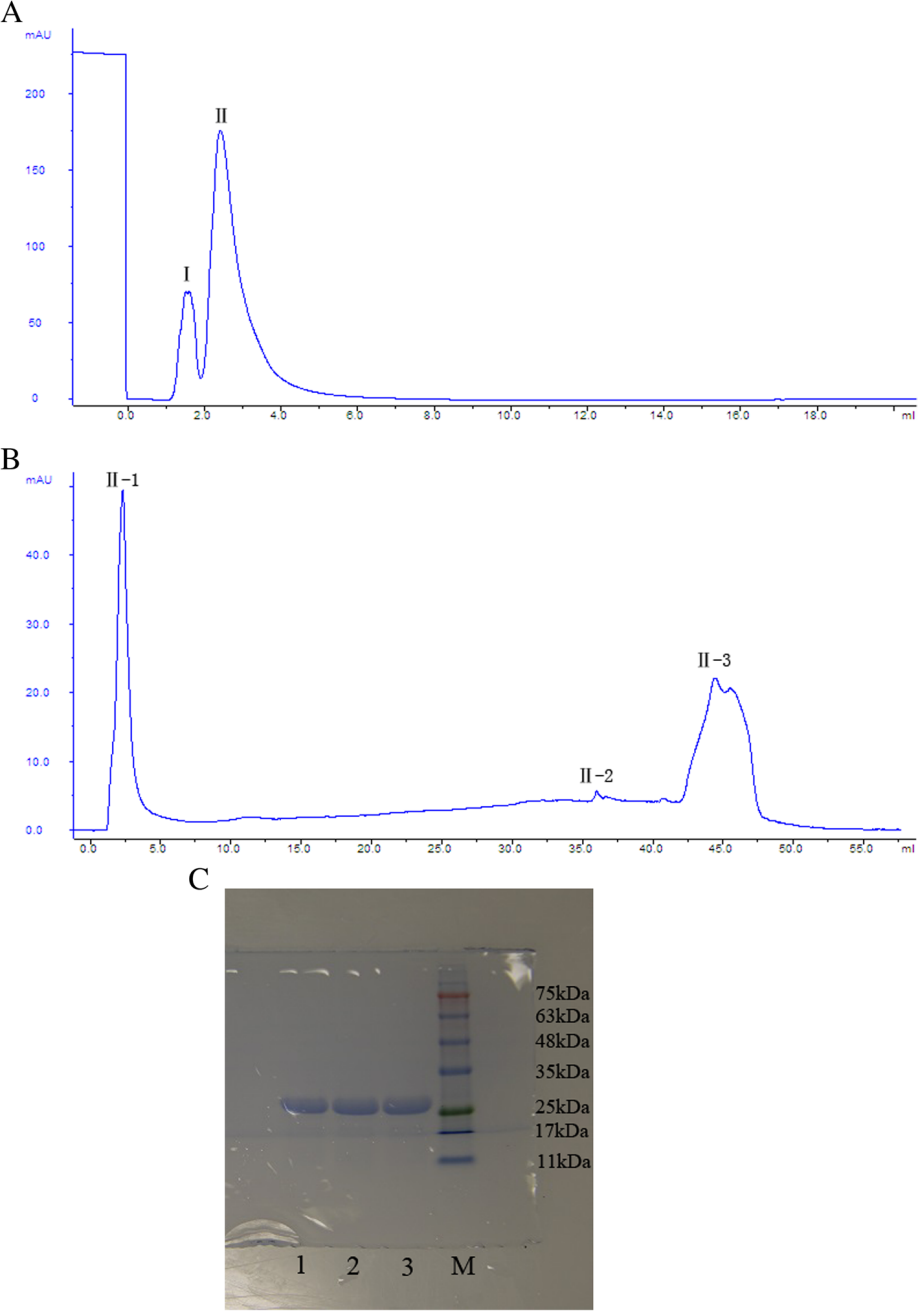
Purification of the protease from strain NJSZ-13 culture filtrates: **A** Elution profile after HIC; **B** Elution profile after IEC; **C** SDS-PAGE of the purified protease from strain NJSZ-13. Lanes 1–3 represent three aliquots of purified protease in parallel; lane M indicates the marker (ColorMixed Protein Marker; Solarbio, Beijing)

The extracellular protease degrading nematode cuticles was purified by 40–60% ammonium sulfate salting-out and exhibited a nematicidal activity of 90.6%. Following HIC, protease and nematicidal activities were assayed in each peak. One peak (II) shown in Fig. 5A was detected as containing protease and nematicidal activities, but for the others, no obvious nematicidal activity was visible. The peak was further purified by IEC (Fig. 5B). Both protease and nematicidal activities were detected in only one peak (II-1). The fraction was pooled and SDS–PAGE of the aliquots showed a highly enriched protein band with a molecular mass about 28 kDa (Fig. 5C). The enzyme activity was measured after each step, and the purification efficiency was shown in Table 2.

**Table 2:** The purification efficiency of the extracellular protease from strain NJSZ-13

Steps	Total protein(mg)	Total enzyme activity(U)	Specific activity(U/mg)	Purification efficiency
Culture filtrate	41.5	1959.6	47.2	1.0
Salting out of ammonium sulfate	16.1	1732.8	107.6	2.3
HIC	7.7	1514.3	196.7	4.2
IEC	4.3	1258.2	292.6	6.2

### Characterization of the protease

**Fig. 6:**
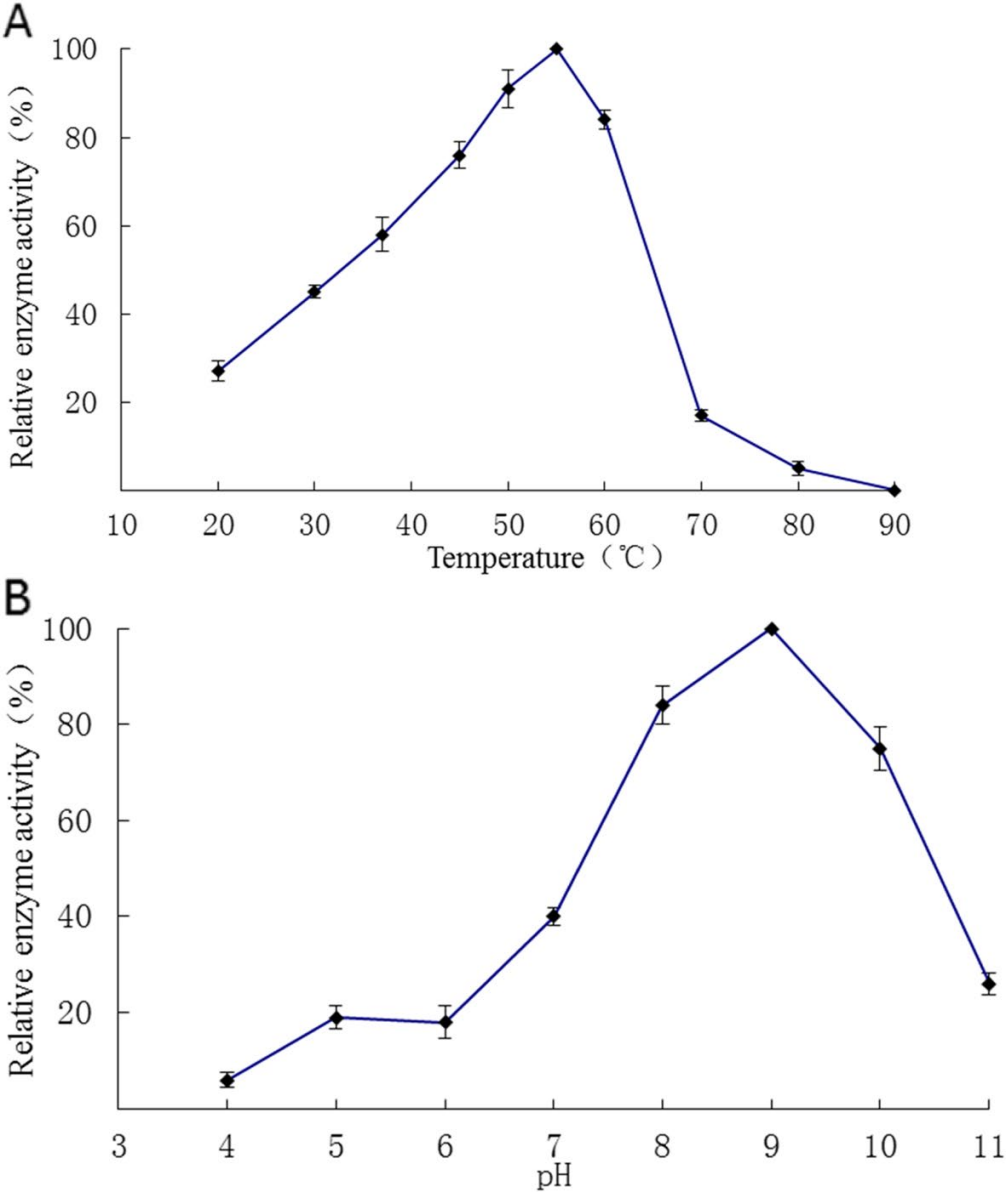
Optimum temperature and pH of the protease. **A**, temperature; **B**, pH. Error bars are standard deviations (*n* = 3)

Temperature studies showed that the enzyme had the highest activity at 55 °C, suggesting that the enzyme was a moderate temperature protease, and the optimum catalytic temperature was about 55 °C. The activity of the enzyme increased gradually when the temperature was in the range of 20–55 °C and decreased gradually in the temperature range of 55–90 °C. When the temperature was over 80 °C，the activity of the enzyme almost lost (Fig. 6A). The enzyme was active over a broad pH range (5.0–11.0) with an optimum at pH 9.0 (Fig. 6B), suggesting the enzyme is an alkaline protease.

The effect of different metal ions on the protease activity is shown in Table 3. 0.5 mM of Na^+^ had a weak activation effect on the enzyme activity, and 5 mM Ca^2+^ and Zn^2+^ showed strong enhancement with the relative enzyme activity of 164.2 and 122.7%, respectively. Li^+^, K^+^, Mg^2+^, Mn^2+^, and Ba^2+^ inhibited the activity of the enzyme and the inhibition becomes stronger with the increase of concentration.

**Table 3:** Effects of metal ions on the NJSZ-13 protease activity

Metal ions	Relative enzyme activity/% (SD)		
	0.5 mM	5 mM	30 mM
LiCl	83.2 ± 2.1	81.7 ± 3.2	56.3 ± 4.7
KCl	90.6 ± 1.3	80.7 ± 1.2	68.8 ± 2.3
NaCl	110.6 ± 2.6	89.3 ± 5.6	72.7 ± 2.7
MgCl_2_	85.3 ± 1.4	69.2 ± 4.3	23.8 ± 2.8
CaCl_2_	102.4 ± 2.8	164.2 ± 4.5	87.7 ± 2.0
MnSO_4_	86.3 ± 2.5	74.7 ± 3.4	61.8 ± 0.4
ZnSO_4_	103.6 ± 3.6	122.7 ± 4.2	56.7 ± 0.9
BaCl_2_	81.9 ± 6.9	72.1 ± 2.6	36.9 ± 2.4
Without metal ions	100 ± 0		

*Note*: SD represents standard deviation (n = 3)

## Discussion

In nature, the natural enemies of the pinewood nematodes include fungi, bacteria, viruses, predatory nematodes, insects, and protozoa. These are important biological factors in the control, balance, and regulation of the nematode population [15–17]. Recently, the use of bacteria against nematode infestations has been the subject of intensive research as bacteria have the advantages of straightforward isolation and rapid growth and reproduction.

In this study, endophytic bacterial strain NJSZ-13 isolated from *Pinus elliottii* in Nanjing was selected for antagonistic effects against the pinewood nematode. In the bioassay experiment, *Bacillus cereus* NJSZ-13 showed significant nematicidal activity. Specifically, over 85% of the nematodes treated with NJSZ-13 were killed within 72 h, and the cuticles of the nematodes were degraded. These results demonstrated the potential of the bacteria in the biocontrol of the pinewood nematode. The genus *Bacillus* has gradually become increasingly important in the study of the mechanism of bacterial infection of nematodes. At present, the species of *Bacillus* with known nematicidal effects are *B. penetrans* [18], *B. firmus* [19], *B. thuringiensis* [20], *B. nematocida* B16 [21], and *B. amyloliquefaciens* FZB42 [22]. The nematicidal mechanisms of these *Bacillus* spp. vary among the species.

In bioassay experiments, the fact that the crude extracellular protein extract exhibited strong toxic activity against the pinewood nematode suggested that the extracellular proteases, as a potential pathogenic factor, may be involved in the infectious process. Similar cuticle destruction was associated with protease activity in nematophageous fungi [23, 24]. Therefore, we purified the protease with nematicidal activity. The purified protease killed about 80% of the tested nematodes and finally destroyed them within 72 h. The results showed that the extracellular protease should be an important cause of death of nematodes and a pathogenic factor of infection.

It is widely known that nematodes have rigid cuticles mainly composed of chitinous microfibers within a protein matrix; in particular, the outer portion of the cuticle is covered by a protein-containing membrane that acts as an effective barrier against infection [25]. Therefore, the extracellular protease that can degrade the cuticle protein membrane of nematodes becomes an important toxin factor to infect nematodes. The roles of extracellular protease, especially serine protease including P32, PII, and pSP-3 have been studied extensively in nematophagous fungi in the infection of nematodes in previous reports [26–28]. Some studies have shown that, during the infection process, protease can promote the growth of host microorganisms by releasing nutrients (mainly amino acids and small peptides), on the other hand, it can promote the penetration of microorganisms or toxins into the host by digesting the host cuticle [29].

In addition, we noted that the mortality of nematodes caused by purified protease (80%, 72 h) was lower than that of crude extracellular protein extract (100%, 72 h), indicating that most (but not all) nematicidal activity is due to extracellular protease. Thus, it cannot be excluded that other pathogenic factors such as toxic peptide which also contribute to the nematode infection of strain NJSZ-13. It is necessary to identify other pathogenic factors to better understand the infection mechanism of strain NJSZ-13 to nematodes.

Biochemical activity assays of the protease showed the maximum protease activity was obtained at 55 °C and pH 9.0, which is an alkaline protease. Among the tested metal ions, Ca^2+^ (5 mM) showed strong enhancement of the protease activity. However, Li^+^, K^+^, Mn^2+^, Ba^2+^, and Mg^2+^ inhibited the protease activity moderately. Ghorbel et al. [30] isolated and purified alkaline protease from *Bacillus cereus* BG1. Ca^2+^ and Mg^2+^ (5 mM) stimulated the protease activity by 450 and 285.5% respectively, which is different from the results in this paper. The research showed that Ca^2+^ can significantly enhance the thermal stability of most alkaline proteases [31], and the mechanism is mainly that under certain temperature conditions, Ca^2+^ can protect alkaline proteases from thermal denaturation, and can also prevent their conformation from changing under high temperature [32]. However, some studies have found that the effect of Mg^2+^ on alkaline protease from different sources is often quite different, which may be related to the amino acid sequence and higher structure of different alkaline protease [33].

To our knowledge, this is the first study utilizing endophytic bacteria isolated from native *Pinus elliottii* to control the pinewood nematode. The details concerning the control effect of the endophytic bacterium NJSZ-13 in nature need further exploration.

## Data Availability

All data generated or analyzed during this study are included in this published article [and its supplementary information files].
